# Microbiologically-Synthesized Nanoparticles and Their Role in Silencing the Biofilm Signaling Cascade

**DOI:** 10.3389/fmicb.2021.636588

**Published:** 2021-02-25

**Authors:** Dibyajit Lahiri, Moupriya Nag, Hassan I. Sheikh, Tanmay Sarkar, Hisham Atan Edinur, Siddhartha Pati, Rina Rani Ray

**Affiliations:** ^1^Department of Biotechnology, University of Engineering & Management, Kolkata, India; ^2^Faculty of Fisheries and Food Science, Universiti Malaysia Terengganu, Kuala Nerus, Malaysia; ^3^Department of Food Technology and Bio-Chemical Engineering, Jadavpur University, Kolkata, India; ^4^Malda Polytechnic, West Bengal State Council of Technical Education, Govt. of West Bengal, Malda, India; ^5^School of Health Sciences, Universiti Sains Malaysia, Penang, Malaysia; ^6^Centre of Excellence, Khallikote University, Berhampur, Ganjam, Odisha, India; ^7^Research Division, Association for Biodiversity Conservation and Research (ABC), Balasore, India; ^8^Department of Biotechnology, Maulana Abul Kalam Azad University of Technology, Haringhata, India

**Keywords:** micronanotechnique, nanoparticles, antibiofilm, quorum-sensing, quorum quencher

## Abstract

The emergence of bacterial resistance to antibiotics has led to the search for alternate antimicrobial treatment strategies. Engineered nanoparticles (NPs) for efficient penetration into a living system have become more common in the world of health and hygiene. The use of microbial enzymes/proteins as a potential reducing agent for synthesizing NPs has increased rapidly in comparison to physical and chemical methods. It is a fast, environmentally safe, and cost-effective approach. Among the biogenic sources, fungi and bacteria are preferred not only for their ability to produce a higher titer of reductase enzyme to convert the ionic forms into their nano forms, but also for their convenience in cultivating and regulating the size and morphology of the synthesized NPs, which can effectively reduce the cost for large-scale manufacturing. Effective penetration through exopolysaccharides of a biofilm matrix enables the NPs to inhibit the bacterial growth. Biofilm is the consortia of sessile groups of microbial cells that are able to adhere to biotic and abiotic surfaces with the help extracellular polymeric substances and glycocalyx. These biofilms cause various chronic diseases and lead to biofouling on medical devices and implants. The NPs penetrate the biofilm and affect the quorum-sensing gene cascades and thereby hamper the cell-to-cell communication mechanism, which inhibits biofilm synthesis. This review focuses on the microbial nano-techniques that were used to produce various metallic and non-metallic nanoparticles and their “signal jamming effects” to inhibit biofilm formation. Detailed analysis and discussion is given to their interactions with various types of signal molecules and the genes responsible for the development of biofilm.

## Introduction

Most chronic infections in humans are found to be caused by biofilm. Biofilm is the syntrophic association of microbial cells that remain adhered to biotic or abiotic surfaces with self-synthesized hydrated polymeric substances (Costerton et al., 1999). The development of biofilm occurs *via* the adherence of planktonic bacterial cells to a surface such as medical devices and prosthetics. It also leads to the development of valve endocarditis, chronic otitis media, cystic fibrosis, and wound-associated infections (Donlan, 2001; Santos et al., 2011; Abidi et al., 2013).

The ability of the bacterial cells to adapt to and monitor various environmental conditions depends on the mechanism of cell-to-cell communication, which is density-dependent and based on contact-associated exchange of chemical substances (Lobedanz and Søgaard-Andersen, 2003; Phelan et al., 2012), chemical signaling (Eberhard et al., 1981), and signaling associated with electrical impulse (Nielsen et al., 2010; Shrestha et al., 2013).

Such a density-dependent communication system in bacteria attributed by small chemical substance (auto inducers) is termed “quorum sensing” (QS). This process was first observed within *Vibrio fischeri* (Nealson et al., 1970) and the term QS was given by Fuqua et al. (1994). The QS machinery comprise of acyl homoserine lactones (AHLs) which are the group of auto inducing peptides that play an important role in causing bacterial pathogenesis. Various virulence factors are produced through QS which include exotoxin A, lection, pyocyanin, and elastase in *Pseudomonas aeruginosa*, whereas protein A, enterotoxin, lipases, hemolysins, and fibronectin were reported in *Staphylococcus aureus* (Yarwood et al., 2004; Carnes et al., 2010). The developed virulence factors help the bacterial cells to evade the host immune system pathogenicity. The formation of biofilm is a multistep process that involves the transcription of various genes with respect to the planktonic form of microbial cells of the same organism (Donlan, 2002). The conversion of the planktonic to its sessile forms results in the enhancement of various chemical substances, resulting in genetic changes within the cells. Thus the sessile micro colonies develop a thick extracellular polymeric substance (EPS) comprising of exopolysaccharides, proteins, extracellular DNA (e DNA), and other polymeric substances that act as a physical barrier around the bacterial cells. This condition results in the maturation of biofilm *via* the process of quorum sensing (QS; Lahiri et al., 2019). The development of biofilm occurs through the mechanism of irreversible attachment of the bacterial cells upon the surface followed by the production of QS molecules, transportation of substances within the biofilm, metabolism of substrate by various sessile micro colonies, development of EPS, and finally the metastasis of the sessile colonies (Lahiri et al., 2019). Although use of antibiotics is the first choice to combat bacterial infection, the rapid increase in antibiotic resistance due to uncontrolled use of antibiotics has become a major health concern (Laxminarayan et al., 2013; Chioro et al., 2015; Zhao et al., 2017; Zhong and Zhao, 2018; Ma et al., 2019; Sarkar et al., 2020). The development of most antibiotics is based on targeting the protein synthesis machinery that in turn results in the destruction of the pathogenic cells (Nikaido, 2009) – precisely the planktonic cells. However, the biofilm-causing sessile cells often remain unaffected and lead to survival of the pathogen.

The traditional approach that was involved in treating biofilm was the combinatorial use of various antibiotics that exhibits various mechanisms of killing. But with the development of antibiotic resistance, conventional drugs are failing to inhibit the formation of biofilm. The development of EPS around the micro colonies prevents the penetration or zero diffusion of antibiotics within the biofilm. The development of EPS around the micro colonies prevents the penetration causing zero or reduced diffusion of antibiotics within the biofilm. Moreover, alterations in the microenvironment within biofilm matrices result in the development of the concentration gradient of metabolites causing reduced or almost no growth of bacteria. It has also been observed that the fluctuation in the microenvironment results in the alteration of nutrient supply, generation of oxidative stress, low availability of water, starvation, and change in temperature which in turn results in the development of adaptive stress responses within the bacterial cells (Singh et al., 2017). It has also been observed that the fluctuation in the microenvironment results in the alteration of nutrient supply, oxidative stress, low availability of water, starvation, and change in temperature which results in the development of adaptive stress responses within the bacterial cells. This is followed by the transformation of the bacterial cells to a highly protected spore-like state, known as persisters, which is also a potent cause of developing resistances towards antibiotics (Stewart, 2002). This issue warrants the exploration of new drugs or drug-like compounds to combat the biofilm.

Recently, the use of various nanoparticles (NPs) has become popular to treat bacterial infections as an alternative to antibiotics. Since NPs follow a totally different mechanism of action to target the bacteria and do not need to penetrate the bacterial cell, new pathways emerged (Wang et al., 2017a).

Microbiologically-synthesized nanoparticles are found to be more advantageous as compared to chemically-synthesized counterparts, as the former does not need very stringent conditions like a pure starting material. The requirement of optimal conditions and clement temperatures (20–30°C) make microbiologically-synthesized NPs more commercially viable (Vaseghi et al., 2018). Moreover, the presence of a biological capping agent on some micro biogenic nanoparticles, as a protective covering against oxidation, agglomeration, and aggregation, offer a higher stability (Durán and Seabra, 2012). Hence, microbiologically-synthesized NPs are often considered to be a better option for antibacterial therapies (Capeness et al., 2019).

Hence, the aim of the present review is to describe the efficiencies of various microbiologically-synthesized nanoparticles as potent antibiofilm agents with special reference to their ability to inhibit quorum sensing through affecting their regulator gene cascade.

## Synthesis of Microbial Nanoparticles

Recently, technological advancements in the field of nanoparticles (NPs) have revolutionized their applicability in healthcare sectors due to the adjustable physico-chemical properties, which include thermal and electrical conductivities, absorption of light, melting point, and enhancement of catalytic activity by altering the surface-to-volume ratio. The field of nanotechnology encompasses the process of synthesizing nano-dimensional particles possessing various shape‐ and size-dependent properties (Rafique et al., 2017). Various types of NPs, including silver nanoparticles (AgNPs), show wide applications in the healthcare domain that include hyperthermia of tumors (Iravani, 2014), delivery of drugs, medical imagining, chemical sensors, catalysis, wireless electronic logic, computer transistors, memory chips, and its antimicrobial efficacy (Das et al., 2014).

Conventionally, NPs are synthesized *via* various physical, chemical, and mechanical processes such as ultra-sonication, radiolysis, microwave, spray pyrolysis, electro spinning, sol-gel method, chemical reduction, and inert condensation methods. But the urgent need for a less time consuming, low cost, high yield, non-toxic, and environment-friendly process has shifted the focus towards greener approaches (Khandel and Kumar-Shahi, 2016; Fang et al., 2019).

Biogenic sources like bacteria fungi and various parts of plants play an effective role in stabilizing the NPs (Durán et al., 2005). The green synthesis of NPs utilizes microbial cells like fungi, yeast, and bacteria as the process can be controlled by manipulating the culture conditions, like nutrient, pH, pressure, and temperature. The microbial system possesses an intrinsic mechanism of synthesizing NPs from metallic salts (Li et al., 2011).

Studies have shown that bacterial cells play an important role in the conversion of heavy metals to metallic NPs. The existence of various types of interactive pathways present within the bacterial cells is responsible for the synthesis of the metallic NPs. Another advantage of implementing bacterial cells is their ability to produce sustainable nanoparticles at a large scale (Fariq et al., 2017). It has also been observed that fungi play a predominant role in the synthesis of NPs *via* both extracellular and intracellular enzymes that are present within cells (Fariq et al., 2017). Enzymes like nicotinamide adenine dinucleotide (NADH)-dependent reductase was responsible for the synthesis of metallic NPs (Guilger-Casagrande and Lima, 2019). Nitrate reductase enzyme and anthraquinones from *Fusarium oxysporum* was responsible for the reduction of silver ions. Another study revealed that extracellular NADH-dependent nitrate reductase from the same fungi and quinolones were used to synthesize AgNPs (Anil Kumar et al., 2007). NADH-dependent oxidoreductase from fungi is also responsible for the synthesis of AuNPs (Kitching et al., 2015). Studies also showed that α-NADH-dependent reductase and nitrate reductase was used for the synthesis of NPs. Due to the presence of larger amounts of biomass in fungi, the yield of NPs is usually higher compared to bacterial cells. Although bacteria are more commonly used in synthesizing metallic NPs, the fungi could be more advantageous due to the presence of mycelia that provide greater surface area for interactions. The amount of enzyme produced by the fungi is higher compared to that of bacteria; thus the rate of conversion of the metallic salts to metallic NPs is faster. The fungal cell wall also played an important role in the mechanism of absorption and the reduction of metal ions for the formation of NPs (Khandel and Shahi, 2018).

The components of fungal cells, like the cell wall, cell membrane, protein, enzymes, and other intracellular components, play a vital role in the synthesis of the nanoparticle. Various parameters like temperature, pH, biomass, and other physical factors participate in regulating the synthesis of metallic NPs like AgNPs. These nanoparticles have various properties which have proven to be useful for human welfare, largely with antimicrobial (antibacterial, antifungal, and antiviral) activities. In fact, the mechanism of biosynthesis of AgNPs by fungi or fungal-based materials does not require any toxic agents during the NP recovery and purification process (Wei et al., 2009). But, like other nanoparticles, mycogenic AgNPs have some disadvantages. The biosafety of the use of AgNPs and their biocompatibility need to be tested prior to application, especially in the field of healthcare. The main obstacle for industrial production of mycogenic metallic NPs lie in the fact that most of the fungal species known for nanoparticle production have been reported to be pathogenic to human and plant. On the contrary, *Trichoderma reesei*, being a nonpathogenic fungus, is now well accepted as an industrially-adapted strain for the production of AgNPs (Dorcheh and Vahabi, 2016). Some other limitations that are associated the fungi-mediated NPs synthesis are higher cost of production and longer time of biosynthesis (Jeevanandam et al., 2016). The advantage of using bacterial species for the synthesis of NPs is to its fast growth and easier mechanism of manipulating genetic expressions (Lovley and Woodward, 1996). The use of bacterial species for the purpose of synthesizing metallic NPs is due to its ability to survive at higher concentrations of metallic ions (Haefeli et al., 1984).

## Mechanism of Synthesis of Microorganism-Assisted Nanoparticles

Processes of both intracellular and extracellular synthesis of nanoparticles (NP) by microorganisms from metals, metal oxides, or metalloids have been well documented in literature (Patil and Chandrasekaran, 2020). The extracellular process involves reduction of metal ions for NPs synthesis by microbial enzymes and proteins, bacterial or fungal cell wall components, or organic molecules present in the culture medium, whereas the intracellular process involves initial electrostatic attraction of metal ions by carboxyl groups of the microbial cell wall, resulting in passage of metal ions through the cells and reduction by intracellular proteins and cofactors to produce NPs (Siddiqi et al., 2018). Biochemical mechanisms involving microorganism-mediated nanoparticle synthesis can be seen as a part of microbial resistance mechanisms for cellular detoxification. This involves alterations in the solubility of inorganic and toxic ions by enzymatic reduction and/or precipitation in the form of nanostructures. Both extracellular and intracellular bio-catalytic synthesis mechanisms have been proposed, which mainly involves oxidoreductase enzymes (e.g., NADH-dependent nitrate reductase, NADPH-dependent sulfite reductase flavoprotein subunit α, and cysteine desulfhydrase) and cellular transporters (Grasso et al., 2019). Nano-dimension materials are biosynthesized within the microorganisms by binding target ions from the surroundings and converting these toxic metal ions into the corresponding element metal through cellular enzymes. Based on the location of synthesis of nanoparticles, it can be classified into intracellular or extracellular. The intracellular method involves transporting ions into the microbial cell to form nanoparticles in the presence of enzymes. The extracellular mode involves trapping the metal ions on the cell surface and reducing ions in the presence of enzymes (Li et al., 2011).

## Microbial Enzymes in Bio Reduction of Metal, Metalloid, and Non-Metal Ions to Nanoparticles

Microbial conversion of metal and metalloids to respective nanoparticles can be accomplished by the extracellular enzymes produced by different bacteria and fungi. Extracellular enzymes, such as nitrate reductase, can help in electron transfer from certain donors (e.g., hydroxyl groups) to Ag^+^ and thus helps in conversion to metallic AgNPs. It has been observed that the functional groups, such as 
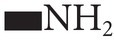
, 
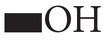
, 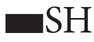, or 
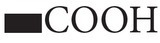
, of microbial proteins help in stabilization of the NPs by providing binding sites to the metal ions followed by its reduction into NPs on the cell wall or in the periplasmic space. In some cases, proteins act as the main reducing or capping agents during the formation and stabilization of NPs. Intracellular enzymes like cytochrome oxidases are also found to help in the reduction of metal ions to NPs *via* electron transfer between cytoplasm components (e.g., NADH/NADPH), vitamins, and organic acids. Intracellular reductase can initiate the biosynthesis and stabilization of NPs in three possible ways: periplasmic reductase can directly reduce M^+^ to M, bio reduction at the cytoplasm or periplasm produces M from M^+^, or bioconversion of M^2+^ to M^+^ in cytoplasm and M formation can occur (Klaus et al., 1999; Mishra et al., 2017; Lv et al., 2018; Siddiqi et al., 2018).

Metalloids such as Te^2+^ and Se^2+^ are harmful to both health and the environment and also involve toxic chemical reductants during their degradation (Presentato et al., 2018). Bio-inspired reductants can be an option for their efficient degradation and decontamination as they produce minimum or no toxic products in the entire degradation process. One such example is the aerobic disintegration of SeO_3_^2−^ by *Actinomycete rhodococcus* into Se-NPs. The reduction of SeO_3_^2−^ to Se-NPs is based on the LaMer mechanism where Se-nucleation seeds were formed, which assemble to form the nanoparticles that precipitated as nano-crystals from the suspension due to higher free energy and lower stability in solutions (Jana, 2015). In another study, Se-NPs were produced both intra-cellularly and extra-cellularly by *Enterobacter cloacae* Z0206 *via* the enzyme fumarate reductase possessing selenite reducing factor. Se-NPs were also produced by microorganisms such as *Citrobacter freundii Y9* (anaerobic synthesis) and *Pseudomonas putida* (aerobic synthesis). In the latter case, it is found that thiol-containing amino acids (such as cysteine) help in the chelation of SeO_3_^2−^ which in turn forms seleno di-glutathione. This again can act as the substrate of glutathione reductase, producing an unstable intermediate Se^0^. Spherical nanoparticles of Se and Te are also formed by microbial species such as *Ochrobactrum* sp. MPV1 and *Stenotrophomonas maltophilia* SeITE02. The detoxification of tellurite to black Te-NPs can be achieved with the help of NADH-dependent reductase (Song et al., 2017; Wang et al., 2017b; Xu et al., 2018).

Intracellular magnetosomes in some bacteria like *Magnetospirillum magneticum* help in the encapsulation of Fe_2_O_3_-NPs in its dissolved form with the help of some multicellular proteins (e.g., ferritin or iron reductase enzymes). Bacterial magnetosomes are organelles of magnetotactic bacteria for geomagnetic navigation and comprises of magnetic nano crystals of magnetic minerals magnetite (Fe_3_O_4_) or greigite (Fe_3_S_4_) that remain surrounded by biological membranes made up of proteins, glycolipids, and phospholipids. The synthesis of magnetosomes is dependent on various environmental conditions, cellular stress, and cell proliferation cycles. The development of the magnetosomes involves transportation of iron outside the bacterial cell membranes *via* the vesicles that are formed, alignment of magnetosomes in a chain, development of crystals, and maturation of the crystals that are being formed (Kuzajewska et al., 2020). The membrane of the magnetosome differs from the plasmalemma on the basis of its composition and provides an appropriate environment for the purpose of biomineralization. This development of magnetosome is a highly controlled process that is regulated by unique protein sets encoded by the magnetosome island (Barber-Zucker and Zarivach, 2017). Supersaturating concentrations of iron also caused nucleation of magnetite at the interface of magnetosome membranes. It has been observed that the formation of vesicles occurs prior to the biomineralization event. Thus, pumping of supersaturating amounts of iron into the vesicles could be performed easier *via* the MamB and MamM proteins, such as in the case of *Magnetospirillum magneticum*. Interactions between the ions of the crystal and the surface proteins help in achieving a better nucleation process. It was also observed that the morphology of magnetite nanoparticles is dependent on the solution chemistry and physical conditions such as super saturation state, iron supply direction, concentration of activator and inhibitor ions or molecules, pH, redox potential, and temperature (Faivre and Schüler, 2008).

These metal oxide nanoparticles use FeCl_3_ as a common precursor. For example, metal oxide nanoparticles of CuO and SnO_2_ were previously made using microorganisms *Morganella morganii* and *Erwinia herbicola*, respectively, utilizing enzymes such as NADH involving redox reactions. The metabolites secreted by the bacteria in the culture broth can induce the reduction and stabilize the newly-formed metal NPs (Srivastava and Mukhopadhyay, 2014; Obayemi et al., 2015).

**Figure 1:**
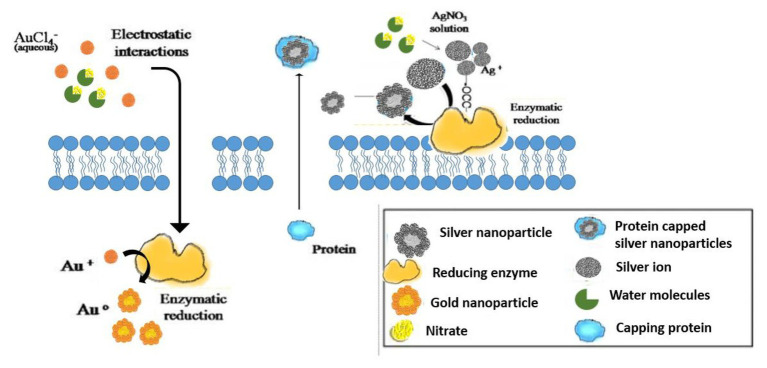
Microbial enzymes in bio reduction of metal, metalloid, and nonmetal ions to nanoparticles.

Nanoparticles of transition metal chalgonides have been synthesized by various researchers. For example, CdS-NPs can be formed by *Moorella thermoacetica* extra-cellularly by the addition of Cd(NO_3_)_2_ in bacterial culture media which helps in the photosynthetic reduction of CO_2_ to acetic acid. CdS-NPs can be produced both extra-cellularly and intra-cellularly by *Desulfovibrio caledoniensis*. It is a three-step process that involves ATP sulfurylase-mediated activation of the anaerobic reduction of sulfate being present within bacteria as well as ferredoxin or NADH-mediated reduction of the resultant adenosine-phosphosulfate (APS) complex to sulfite followed by assimilatory or dissimilatory sulfite reductase which reduced sulfite to sulfide. PbS nano crystals can also be biosynthesized by controlling the concentration of poly-etheneglycol in the *Clostridiaceae* sp. where SO_4_^2−^ is first reduced to S^2−^ by the sulfate-reducing bacteria, and then S^2−^ gradually combined with Pb^+2^ to precipitate as PbS-NPs (Qi et al., 2016; Yue et al., 2016). Studies have also shown that grapheme-associated highly dispersed Pd-Ag bimetallic NPs can be synthesized using *Shewanella oneidensis* MR-1(Han et al., 2019). The conversion of graphene oxides to grapheme nano sheets can be achieved through the use of crude polysaccharides obtained from *Pleurotus flabellatus* (Dasgupta et al., 2017; Figure 1).

## Microbial Exopolysaccharides for Synthesis of Nanoparticles

Exopolysaccharides (EPSs) from bacterial cells are produced extra-cellularly and play vital roles in surface adherence and cell-cell communication. EPSs possess the ability of reducing metal ions to produce nanoparticles and also help in the stabilization of the NPs by acting as a capping agent. Therefore, the EPSs are used as an alternate choice for microbiological production of numerous metal nanoparticles. Bacterial EPSs mainly comprise of carbohydrates such as D-glucose, L-fucose, D-mannose, D-galactose, N-acetyl-D-glucosamine, N-acetyl D-galactosamine, and non-carbohydrate components which are responsible for the anionic nature to the EPSs. These organic groups tend to increase the lipophilicity of the EPSs and directly influence their interaction with cations such as metal ions. Metal ions in contact with EPS are chelated and then reduced and stabilized by various functional groups *via* electrostatic bonds. For example, oxidation of 
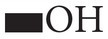
 groups to form 
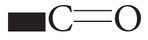
 groups and oxidation of 
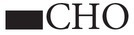
 groups to form 
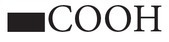
 groups play an important role during metal nanoparticles synthesis. The polymeric structure of EPSs create a network by 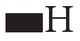 bonding in which nanoparticles stabilize with subsequent preventions of their agglomeration and precipitation (Escárcega-González et al., 2018). Various types of functional groups that are associated with EPS of both Gram positive and Gram negative acts as reductive and stabilizing agents for the purpose of synthesizing NPs with the capping and chelating processes (Emam and Ahmed, 2016). This helps in the regulation of size, particle dispersion, and shape of the NPs (Kanmani and Lim, 2013). The ability of the NPs to develop broader applicability is due to the muco-adhesion properties that help in the development of non-specific protein receptor recognition by the NPs (Kanmani and Lim, 2013). In previous research it was revealed that structurally known EPS from succinoglycanbacteria were used for the synthesis of AgNPs. It is a polymeric substance produced by *Sinorhizobium meliloti* that helped in the reduction of the metal by inducing oxidation of the aldehyde group to a carboxyl group with the help of nucleophilic insertion (Kwon et al., 2009). Curdlan is another type of EPS comprising of (1, 3)-β-D-glucan repeated units that are joined by β-(1, 3)-glycosidic bonds and are produced predominantly by *Alcaligenes faecalis*, *Rhizobium* sp., and *Agrobacterium* sp. These are used for the purpose of synthesizing and stabilizing NPs (Zhang and Edgar, 2014). Curdlan, a water insoluble polymeric substance, can be carboxylated or oxidized to curdlan derivatives for the purpose of synthesizing NPs. Leung et al. (2010) synthesized AgNPs with the help of carboxymethylated-curdlan. The persistence of the negatively-charged hydroxyl group and carboxyl groups resulted in better reduction of the silver ions. Dextran is another predominant component of EPS that helps in the synthesis of grapheme NPs (Hu et al., 2016). Chemically, dextran is a multifaceted branched glucan comprising of glucose residues that remain interlinked with α-(1,6) glycosidic linkages and are predominantly produced by certain groups of lactic acid bacteria like *Leuconostoc mesenteroides* and *Streptococcus mutans*. Bankura et al. (2012) synthesized size-controlled AgNPs using the aqueous solution of dextran which acted as both reductant and stabilizer.

Au NPs can also be green synthesized using *Cupriavidus metallidurans* and *Delftia acidovorans* Au nuggets can be obtained from the bacterial biofilms (Johnston et al., 2013). Experimental observations revealed that nano particulate Au can inhibit biofilm formation (Reith et al., 2010). Au NPs bring about inhibition of biofilm by altering the surface chemistry and hydrophobicity as well as interacting with lipids and proteins that are present on the bacterial cell membrane (Ikuma et al., 2015). This alters the penetration of the NPs within the biofilm. The ability of NPs to penetrate the biofilm depend on the charge existing on the surface, size of the NPs, and the chemistry and concentration of NPs (Ikuma et al., 2015). The penetration of the NPs is followed by its interaction with the structural components of the biofilm, causing the disintegration of the biofilm (Qayyum and Khan, 2016; Pinto et al., 2019). It has been further observed that modified groups of the Au NPs can increase its inhibition potential either on single or multiple types of cells present within the biofilm. Various types of forces, like Van der Waals, hydrogen-bonds, electrostatic, and hydrophobic interactions, can effectively inhibit the biofilm (Yu et al., 2018).

## Microbial Bio Surfactants for Synthesis of Nanoparticles

Bio surfactants are microbial surface-active amphiphilic molecules produced mainly by bacteria, fungi, and yeasts. Their hydrophilic moiety consists of carbohydrates, cyclic peptides, amino acids, carboxylic acids, or phosphates and the hydrophobic moiety comprises mainly of long-chain fatty acid or hydroxyl fatty acid. These are divided to two types *viz*. low-molecular-weight surface active agents (LMW) such as glycolipids, lipopeptides, and phospholipids and high molecular-weight polymers (HMW) mainly referred to as bio emulsifiers such as emulsan (Pati et al., 2020). They can also be classified as: (i) hydroxylated and cross-linked fatty acids (mycolic acids); (ii) glycolipids (rhamnolipids); or (iii) lipopolysaccharides. Bio surfactants can act as excellent capping agents during the synthesis of metallic nanoparticles *via* biogenic processes (Płaza et al., 2014). Their mechanism of action involves adsorption onto metallic nanoparticles, surface-stabilizing the nanoparticles, and preventing subsequent aggregation, thereby helping in stabilization process (Kiran et al., 2011; Gahlawat and Choudhury, 2019). Biosurfactants are the groups of amphipathic molecules possessing both hydrophobic and hydrophilic moieties creating partitions at the interface between fluid phases possessing various degrees of hydrogen bonding and polarity (Rodrigues et al., 2006). The micro emulsions, i.e., the water-soluble droplets that are present within, act as a micro-reactor. The increase in the concentration of the surfactants results in a decrease in the size of droplets, thereby reducing the particle size. The presence of water plays an important role in regulating the size and the morphology of the NPs. The size of the particle and mono dispersity is dependent on the molar ratio (R) of the water (Han et al., 2008).

## Microbial Synthesis of Nanoparticles Through Bio Mineralization

Some microorganisms possess the unique property of mobilizing/immobilizing the metal salts by reducing them into metal ions which precipitate within or outside the microbial cells. They, with the help of efflux pumps, accomplish the complexation and inactivation of metals, with their subsequent precipitation by changing the oxidation state of the metals *via* redox reactions.

For example, gold (I)-thiosulfate enters *Acidithiobacillus thiooxidans* cells metabolically and are broken down to Au(I) and thiosulfate (S_2_O_3_^2−^) ions. Thiosulfate acts as an energy source while Au(I) reduces itself to elemental gold intracellularly. These elemental gold precipitates inside the bacterial cells to form NPs during the late stationary growth phase and are released from the cells later on. Finally, the gold particles in the bulk solution are grown into micrometer-scale wire and octahedral gold (Lengke and Southam, 2005).

In another study, it was proposed that the sulfate-reducing bacteria bring about the reduction of the gold (I)-thiosulfate complex through three possible pathways: iron sulfide formation, localized reducing effects, or a metabolic pathway. The first process involves the adsorption of gold (I)-thiosulfate on the surfaces of iron sulfide freshly formed by sulfate-reducing bacteria producing elemental gold. The second process involves localized reducing conditions causing the gold (I)-thiosulfate complex reduce to hydrogen sulfide (HS^−^) by sulfate-reducing bacteria which was released through the outer membrane pores resulting into precipitation of elemental gold. The third process involved decomplexation of gold (I)-thiosulfate complex to Au(I) and thiosulfate ions which are later released from the cells (Lengke and Southam, 2005; Lengke et al., 2006).

## Microbial Synthesis of Magnetic Nanoparticles

The bacterial magnetic nanoparticle (BMP) formed from magnetotactic bacteria (MTB), known as magnetosomes, which are a type of magnetic nanoparticle, has a lot of possibilities in nano biotechnology (Vargas et al., 2018). These are intracellular magnetic particles comprising of oxides and sulfides of iron within the bacterial cell that act as a bacterial compass needle helping bacterium to migrate along oxygen gradients in aquatic environments, under the influence of the Earth’s geomagnetic field. BMPs have the ability to disperse in aquatic medium and are usually carried by phospholipid vesicles.

Bio mineralization of BMPs occurs *via* multiple steps; the first step involves a GTPase-mediated invagination of the cytoplasmic membrane followed by assembly into a linear chain along the cytoskeletal filaments. The second step involves trans membrane iron-transporter-mediated accumulation of ferrous ions inside the vesicles. The final step involves magnetite crystal nucleation by triggering BMP proteins which includes the accumulation of supersaturating iron concentrations, and partial reduction and dehydration of ferrihydrite to magnetite (Arakaki et al., 2008).

In another study, magnetite was synthesized by *Shewanella oneidensis* involving both passive and active methods. Active utilization of ferrihydrite as a source of electron acceptor to form Fe^2+^in a high pH environment followed by localized conversion of Fe^2+^ and Fe^3+^ near negatively charged cell walls leads to super saturation, causing magnetite to precipitate (Li et al., 2011). The membrane controls the particle size, crystallization, and particle morphology in the particle size, crystallization, and particle morphology. The phospholipids bilayer can entrap the bacterial nanoparticles and contains about 20–40 species of membrane proteins (Grünberg et al., 2004).

The BMPs used in the nano biotechnology and nano medicine are mainly extracted from species of *Magnetospirillum gryphiswaldense* MSR-1 and *Magnetospirillum magneticum* AMB-1, although there are other species of MTB that can be cultivated (Chen et al., 2016).

## Microbial Synthesis of Stable Quantum Dot Nanoparticles

Recently, fluorescent or quantum dots (QDs) nanoparticles have been widely used in several biological, biomedical, optical, and optoelectronic applications such as biosensors, photovoltaics, optoelectronics, transistors, oil exploration, biomedicine, imaging, and solar cells due to its unique size-dependent properties. Their increased utility is due to their biocompatibility and lesser toxic by-product generation during its synthesis, indicating a pathway to greener technology. So far, synthesis of CdS, CdSe, and CdTe QDs involved the use of harmful chemicals such as bidentate thiols [e.g., dithiothreitol (DTT), mercaptosuccinic acid (MSA), mercaptopropionic acid (MPA)], and ligands with different functional groups (amino, hydroxyl, and carboxylic acid, among others). Researchers have isolated bacteria that are cadmium‐ and tellurite-resistant Antarctic bacteria *Pseudomonas* (eight isolates), *Psychrobacter* (three isolates) and *Shewanella* (one isolate) capable of synthesizing CdS and CdTe QDs when exposed to toxic oxidizing heavy metals like Cd and Te with a time-dependent change in fluorescence emission color (Plaza et al., 2016). The CdSe nanoparticles are one of the examples that exhibit fluorescent tags. Cui et al. (2009) reported intracellular synthesis of CdSe nanoparticles in *S. cerevisiae* using genetic engineering techniques. The genes involved in glutathione biosynthesis, namely *GSH1*, *GSH2*, and *GLR1*, become silent in yeast and after interaction with inorganic ions resulted in a significant reduction in fluorescence, which in turn was proportional to the amount of CdSe nanoparticle synthesis. It was found that Na_2_SeO_3_ was reduced to selenocysteine (Cys-Se)_2_, a complex of selenium-containing cysteine, which after the addition of CdCl_2_ generated CdSe nanoparticles. In another work by Bruna et al. (2019), CdS QDs by polyextremophile halophilic bacteria *Halobacillus* sp. DS2 were synthesized with increased tolerance to NaCl (Bruna et al., 2019). A tunable ternary CdSAg QD involving cation exchange were synthesized by Órdenes-Aenishanslins et al. (2020). Nanoparticles were also produced within bacterial cells extra-cellularly *via* exposure to cysteine and CdCl_2_ in a reaction. This reaction was dependent on S^2−^ generation mediated by cysteine desulfhydrase enzymes and utilized cellular biomolecules to stabilize the nanoparticle.

## Microbial Organic Particles for Nanoparticle Synthesis

Bacterial cellulose (BC) are used to form nano fibers and to instill a bactericidal property in the nano fibers. A combination of bactericidal chitin (Ch) with bacterial cellulose (BC) nano fibers was developed to form a nanocomposite of BC-Ch in a process that is considered a green approach. *Acetobacter aceti* was also fed with Ch_79d_ to biosynthesize bio-BC-Ch_79d_ nanocomposites to produce 50–100-nm-wide nanofibrils (Butchosa et al., 2013).

A number of microbial components are found to be involved in the formation of nanoparticles (Table 1), which are found to act as potent antibiofilm agents through inhibition of the quorum-sensing process.

**Table 1:** Contribution of microbes in the formation of nanoparticles.

Classification	Microorganism	Microbial component	Raw material	Element used	Nanoparticle size (nm)	Morphology	Synthesis location	References
Bacteria	*Lactobacillus rhamnosus*	EPS		Ag	10	Spherical, triangular, rod, and hexagonal	Extracellular	Kanmani and Lim, 2013
	*Bacillus licheniformis* Dahb1	EPS		ZnO	100	Hexagonal	Extracellular	Abinaya et al., 2018
	*Brevibacterium casei* MSA19	Biosurfactant		Ag			Extracellular	Kiran et al., 2010
	*Bacillus amyloliquifaciens* KSU-109	Biosurfactant	Surfactin	CdS			Extracellular	Singh et al., 2011
	*Pseudomonas aeruginosa* BS-161R.	Biosurfactant	Rhamnolipid	Ag			Extracellular	Kumar et al., 2010
	*Bacillus cereus*			Ag	20–40	Spherical	Extracellular	Sunkar and Nachiyar, 2012
	*Kocuria flava*			Cu	5–30	Spherical	Extracellular	Kaur et al., 2015
	*Pseudomonas aeruginosa* JP-11			CdS	20–40	Spherical	Extracellular	Raj et al., 2016
	*Shewanella loihica* PV-4			Pd and Pt	2–7	Spherical	Intracellular	Ahmed et al., 2018
	*Ochrobactrum* sp. MPV			Te		Spherical, rod	Intracellular	Zonaro et al., 2017
	*Bacillus cereus*			Ag	20–40	Spherical	Extracellular	Sunkar and Nachiyar, 2012
	*Escherichia coli*			Ag	8	Spherical	Extracellular	Mahanty et al., 2013
	*Geobacillus* sp.			Au	5–50	Quasi hexagonal	Extracellular	Correa-Llantén et al., 2013
	*Bacillus subtilis*			Ag	20–25	Spherical	Extracellular	Srinath et al., 2018
	*Nocardiopsis* sp. MBRC-1			Ag	45	Spherical	Extracellular	Manivasagan et al., 2013
	*Pseudomonas fluorescens*			Au	50–70	Spherical	Extracellular	Rajasree and Suman, 2012
	*Serratianematodiphila*			Ag	10–31	Spherical crystalline	Extracellular	Malarkodi et al., 2013
	*Shewanellaoneidensis*			U	150		Extracellular	Satyanarayana Reddy et al., 2010
	*Magnetospirillummagneticum*			Fe_3_O_4_	10–60	Cuboidal, rectangular and spherical NPs	Intracellular	Obayemi et al., 2015
	*Shewanellaoneidensis* MR-1			CdSe	3.3		Intracellular	Tian et al., 2017
	*Escherichia coli*			CdTe	62		Extracellular	Kominkova et al., 2017
	*Clostridiaceae* sp			PbS	Cubic NPs	50	Extracellular	Yue et al., 2016
	*Erwinia herbicola*			SnO_2_	15–45	Spherical andtetragonal NPs	Extracellular	Srivastava and Mukhopadhyay, 2014
	*Serratia marcescens*			Bi	<150	Irregular	Intracellular	Nazari et al., 2012
Actinomycetes	*Streptacidiphilus durhamensis*			Ag	8–48	Spherical		Buszewski et al., 2018
	*Streptomyces griseoruber*			Au	5–50	Spherical, hexagonaland triangular		Ranjitha and Rai, 2017
	*Streptomyces xinghaiensis* OF1			Ag	5–20	Spherical		Wypij et al., 2018
	*Rhodococcus* sp. NCIM2891			Ag	10–15	Spherical		Otari et al., 2014
Fungi	*Penicillium diversum*			Ag	10–15	Spherical		Ganachari et al., 2012
	*Fusarium oxysporum* JT1			Au	22	-		Thakker et al., 2013
	*Trichoderma harzianum*			CdS	3–8	Spherical		Bhadwal et al., 2014
	*Aspergillus terreus*			ZnO	28–63	Spherical		Baskar et al., 2015
	*Colletotrichum* sp.			Al_2_O_3_	30–50	Spherical		Suryavanshi et al., 2017
Yeast	*Rhodosporidium diobovatum*			PbS	2–5	Spherical		Seshadri et al., 2011
	*Saccharomyces cerevisiae*			Ag, Au Nanoplates	2–20	Spherical, hexagonal and triangularnanoplates		Korbekandi et al., 2016; Yang et al., 2017
	*Pichia kudriavzevii*			ZnO	10–60	Hexagonal wurtzitestructure		Moghaddam et al., 2017
	*Rhodotorula glutinis*			Ag	15	Spherical		Cunha et al., 2018
Virus	Tobacco mosaic virus (TMV)			Pd, Au	3–4, 5	Carbon nanotubes, spherical		Kobayashi et al., 2012; Fan et al., 2013
	M13 virus			TiO_2_	20–40	Mesoporous nanowires		Chen et al., 2013
	Hepatitis E virus			Nanoconjugates	27–34	Icosahedral		Chen et al., 2018
	Potato virus X			Nanocarriers	13	Helical		Le et al., 2017
Algae	*Sargassum muticum*			ZnO	0–57	Hexagonal		Sanaeimehr et al., 2018
	*Gelidium amansii*			Ag	27–54	Spherical		Pugazhendhi et al., 2018
	*Laminaria japonica*			Ag	31	Spherical to Oval		Kim et al., 2016
	*Cystoseira baccata*			Au	8	Spherical		González-Ballesteros et al., 2017
	*Chlorella vulgaris*			Pd	5–20	Spherical		Garole et al., 2019
	*Spirogyra varians*			Ag	35	Quasi spherical		Salari et al., 2016
	*Chlorella vulgaris*			Au	2–10	Spherical		Annamalai and Nallamuthu, 2015

## Mechanism of Quorum Sensing

**Figure 2:**
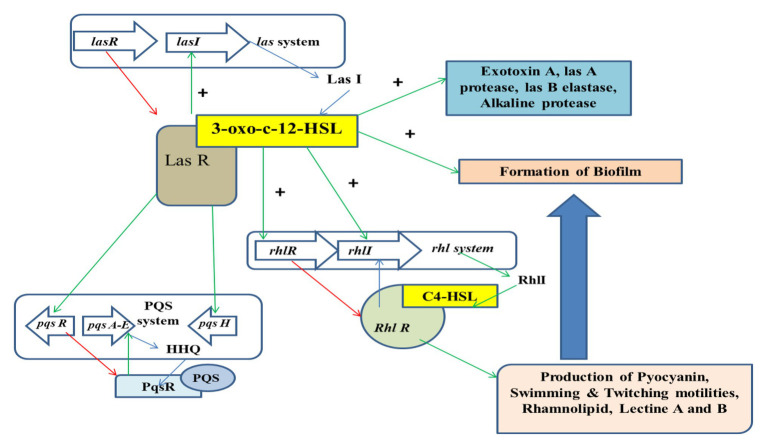
Regulation of the mechanism of QS.

The process of QS is the mechanism of interaction between the bacterial cells *via* the production of extracellular chemicals known as Auto inducers (AIs). This mechanism helps in the process of synchronizing the bacterial cells and their various expressions to respond to the changes of the environment. This process is observed in both Gram-positive and Gram-negative bacterial cells. Studies have shown that Gram-negative bacterial cells possess three major groups of Ais, whereas the Gram positive bacterial cells communicate with the help of auto inducing peptides (AIPs; Raffa et al., 2005). The mechanism of QS can be inhibited by the process of quorum quenching (QQ; Dong et al., 2002). Various mechanisms are involved in the process of QQ that comprise of competitive inhibition and cleavage of the QS signals. This predominantly brings about the inhibition of QS. The chemical that inhibits the mechanism is referred to as Quorum sensing inhibitor (QSI), whereas the enzymes involved in such inhibition are QQ enzymes. It has often been noticed that QQ enzymes can inhibit QS by targeting AHLs. Several classes of enzymes are capable of degrading AHL signal, such as acylases and oxido reductases that are predominantly of a bacterial origin. It has been also observed that the half-life of AHL is dependent on pH and temperature (Yates et al., 2002; Delalande et al., 2005). There are two novel AHL enzymes, namely *N*-acyl homoserine lactonase (AiiA) and esterase (Est), that can be isolated from *Altererythrobacter* sp. S1-5 (Wang et al., 2019). The former enzyme can hydrolyze and inactivate a variety of acyl homoserine lactones (AHLs). Quorum sensor molecules involved in bacterial quorum sensing (QS) were extracted and purified from *Bacillus* sp. 240B1. After its covalent immobilization onto magnetic nanoparticles (MNPs), the quorum quenching ability of r-AiiA-MNP nano biocatalyst was evaluated and was found to be effective in inhibiting QS (Beladiya et al., 2015). The las system comprises of the *lasR* regulatory gene that codes for the Las R protein, while lasI synthase is regulated by *lasI* gene which is associated with the synthesis of signaling molecule of the AHL family, i.e., 3-oxo-C12-HSL. The LasR/3-oxo-C12-HSL is responsible for activating the virulence gene. The *rhl* system comprises of *rhlR* and *rhlI* genes. These further results in the activation of the las system that is responsible for the production of pyocyanin, rhamnolipids, and swarming motilities. The rhl system is regulated by the las system. PQS intermediates between the two other systems. The PqsA_E acts as a precursor for 2-heptyl-4-quinolones (HHQ) and regulates the conversion of HHQ to 2-heptyl-3-hydroxy-4-quinolone (PQS; Figure 2; Carette et al., 2020).

## Mechanism of Quorum Sensing in Gram-Negative Bacteria

The Gram-negative bacterial cells communicate *via* signaling molecules, termed as auto inducers (AI), such as acetyl homoserine lactone (AHL) and other chemical molecules whose synthesis is dependent on S-adenosylmethionine (SAM; Walker et al., 2011). SAM acts as an amino acid subtract required for the synthesis of acyl homoserine lactones (Whitehead et al., 2001). A study showed that *E. coli* comprising of plasmid associated *lux I* requires the presence of SAM for the synthesis of *N*-(3-oxooctanoyl)-L-homoserine lactone (Hanzelka and Greenberg, 1996).

The AI produced by the bacterial cells can easily diffuse through the outer layer of the cell membrane. Enhancement of AIs is observed during high cell density (HCD) and thus regulates the transcriptional factors for the genes that are associated with the process of QS. Various types of signaling molecules are associated with the Gram-negative bacterial cells like 3-hydroxy-palmitic acid from *Ralstonia solanacearum* and 2-heptyl-3-hydroxy-4-quinolone from *P. aeruginosa* (Flavier et al., 1997). The latter is an opportunistic nosocomial disease-causing organism remaining associated with infections like cystic fibrosis (CF), lung infection, and various types of dermal and burn wound infections (Ammons et al., 2009). These Gram-negative bacteria comprise of three major QS circuits. One such circuit is the *lasI* encoding protein LasI that is associated with the production of auto inducer as well as the gene *LasR* that in turn encodes for the transcriptional activator LasR. Another profound QS circuit comprises of gene *rhlI*, associated with the synthesis of autoinducer-N-(butonyl)-L-Homoserine lactone and rhlR that is responsible for the transcriptional activator RhlR (Pearson et al., 1994, 1995). The third QS circuit which is also observed in *P. aeruginosa* is related to alkyl quinolones, especially 2-heptyl-3-hydroxy-4-quinolones that are predominantly regulated by *pqsABCDEH* and *PqsR* regulator genes (Pesci et al., 1999).

## Mechanism of Inhibition of Quorum Sensing by Micro Biogenic NPs

Information pertaining to the inhibition in the mechanism of QS by NPs are very limited as only a few studies have been performed; this therefore remains a somewhat underexplored field despite being interesting. NPs act as potent inhibitors of QS by affecting the mechanism of cell-cell communication or inhibition of the signals associated with the mechanism of QS, thereby hindering the synthesis of various types of the signaling molecules and preventing the formation of molecule-receptor complex. This in turn stops the signal transduction cascade (Sadekuzzaman et al., 2015) Silver nanoparticles (AgNPs) have been used as QQ agents due to their strong antimicrobial activity (Castellano et al., 2007; Chen and Schluesener, 2008).

**Figure 3:**
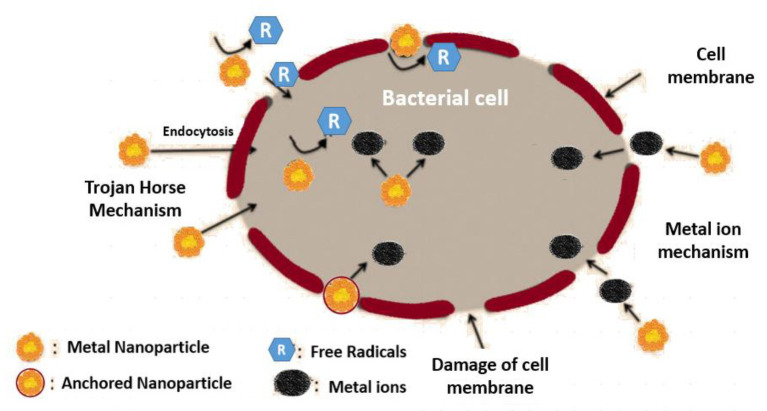
Mechanism of NPs to bring about damage to the bacterial cells.

The scientific communities have shown their interest in the use of AgNPs due to their broad range of antimicrobial activities (Kim et al., 2007; Lara et al., 2011; Brandt et al., 2012) and the convenience during application for their physico-chemical properties and surface area to volume ratio (Kim et al., 2007; Figure 3). Various other types of NPs, like AuNPs, TiO_2_, SiO_2_, and ZnO, from microbial sources possess the efficacy of inhibiting the QS cascade and thus inhibit the formation of the biofilm (Shah et al., 2008; Samanta et al., 2017; Al-Shabib et al., 2018).

## NP-Associated Inhibition of Quorum Sensing Cascade

The growth of the sessile communities of bacteria within the biofilm can only be checked by quorum sensing (QS), a bacterial cellular communication system that is a non-cytotoxic process (Høiby et al., 2010). The success in the treatment of any kind of chronic infections is devising an efficient mechanism of delivering the drug molecules up to the target cells. NPs have played a pivotal role in the mechanism of inhibiting the process of QS, and thus could inhibit biofilm formation.

The nano materials possess dimensions at the scale of nanometers possessing chemical and physical properties different from that of the bulk materials (Wang et al., 2017a). NPs are proven to be the most accepted drug delivering vehicle, possessing the ability to inhibit the growth of the microbial cells and thus can fight against pathogenic organisms. NPs show various mechanisms pertaining to the inhibition of biofilm and microbial growth. Various studies were conducted to predict the probable inhibition mechanism of microbial growth by the NPs. It was observed that ZnO NP has the ability of inhibiting the NorA efflux pump present in *S. aureus* (Banoee et al., 2010). It has been further observed ZnO NP, along with the antibiotic ciprofloxacin, could enhance the zone of inhibition by 22–27% of *E. coli* and *S. aureus*, respectively (Banoee et al., 2010). Studies also showed that iron oxide NPs coated with polyacrylic acid help in inhibiting *Mycobacterium smegmatis* by enhancing the efflux inhibition (Padwal et al., 2015).

## Inhibition of Quorum Sensing by Micro Biogenic Silver Nanoparticles

**Figure 4:**
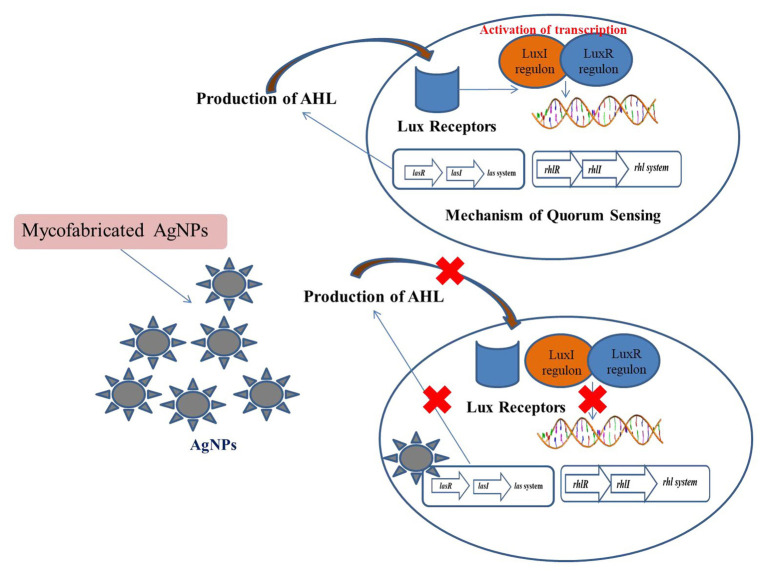
Mechanism of inhibition of QS by myco-fabricated Ag NPs.

The antimicrobial potential of AgNPs has made it an important therapeutic agent. But advancements in the era of antibiotics has resulted in the minimization of the use of silver (Castellano et al., 2007; Li et al., 2009). The presence of wide spectrum antimicrobial activities by AgNPs due to its high surface area to volume ratio has been a key factor in its success (Kim et al., 2007; Lara et al., 2011; Brandt et al., 2012). Various studies have been conducted that showed that silver composites and silver have significant roles as antimicrobial agents (Panáček et al., 2006; Naik et al., 2013). AgNPs also act as potent bactericidal agents against *S. aureus*, *P. aeruginosa*, and *E. coli* (Khurana et al., 2016). Literature has shown that AgNPs can be used as potent anti-QS agents, thus hindering the formation of the biofilm and production of violacein by *C. violaceium* (Jagtap and Priolkar, 2013). It has been further observed that green-synthesized NPs played a key role in controlling infections associated with microbes. Studies have revealed that Ag NPs have the potentiality of blocking the synthesis of signaling molecules by inhibiting LasI/Rhl I synthase. Ag NPs had the potential of inhibiting the QS of *P. aeruginosa* (Ali et al., 2017). In-silico studies comprising of molecular docking revealed that Ag NPs have the potency of locking the active sites of various proteins comprising of LasI or RhlI synthase along with their surrounding residues being present. Ag NPs effectively block the active sites and thus efficiently inhibit the mechanism of QS. Ag NPs possess the ability of inhibiting the QS-genes by blocking the transcriptional regulatory proteins which inactivates the LasR or RhlR system. Ag NPs also possess the ability to act effectively as anti-QS agents by inhibiting signaling molecules like LasI and RhlI. Studies have also shown that micro-fabricated forms of Ag NPs synthesized from *Rhizopus arrhizus* metabolites inhibit the QS mechanism of *P. aeruginosa* (Singh et al., 2015b). It was observed that micro fabricated Ag NPs were able to bring about a marked reduction in the production of signaling molecules at a concentration of 0-25 μg/ml. It was further observed that these micro-fabricated NPs were able to bring about a reduction of 79–84% of *lasA* and *lasB* genes’ expression and down regulated these genes. The expression of the targeted QS genes, like *lasA*, *lasB*, *lasI*, *lasR*, *rhlI*, *rhlR*, *rhlA*, *phzA1*, and *fabH2*, is activated by the AHL-LasR complex. The down regulation of the QS genes was achieved by the myco-fabricated Ag NPs. Its mechanism against various virulence factors such as LasB ealastase, LasA protease, rhamnolipid, and pyocyanin occurred *via* phzA1, rhlA, and lasAB operons. The production of AHL is enhanced by *rhlR* via and the signal cell receptor RhlR. Similarly, RhlR is also associated with the activation of fabH2 and rhlAB operons. Thus myco-fabricated Ag NPs help in the down regulation of *rhlR* and thereby decrease the production of RhlR (Singh et al., 2015a; Figure 4).

## Inhibition of Quorum Sensing Using Micro Biogenic Gold Nanoparticles

In recent times, the use of Au NPs have attracted various researchers due to their catalytic properties that are largely used in the field of diagnostics and biologics (Mesbahi, 2010; Jain et al., 2012; Giasuddin et al., 2013). The wide applicability of Au NPs is due to its simple mechanism of synthesis, convenience to use, and relatively low toxicity in comparison to the other types of nano materials in use (Capek, 2014). Au NPS showed efficient antimicrobial activity against various types of microbes like methicillin-resistant *S. aureus* (MRSA), *Salmonella typhi*, and *Bacillus Calmette-Gu’erin* (Zhao et al., 2010; Lima et al., 2013; Bindhu and Umadevi, 2014). Studies also revealed that acyl homoserine lactone lactonase protein associated with An NP help in inhibiting QS of *Proteus* sp. (Vinoj et al., 2015). These NPs were also able to degrade N-hexanoyl-L-homoserine lactone with the help of N-acyl homoserine lactonase that was present on the surface of the NPs. The enzyme also helps in degrading the moiety associated with acyl homoserine lactone and brings about the conformational changes of the signaling molecule, thus preventing the binding with LuxR transcriptional regulator resulting in the inhibition of QS (Kaufmann et al., 2005; Bai et al., 2008). They were also able to inhibit the metabolic activities and production of EPS, preventing the formation of biofilm and changing the hydrophobicity of the bacterial cells (Samanta et al., 2017). The Au NP produced from the mycelium of *Laccaria fraternal* was responsible for stabilizing the NPs which in turn plays an important role in the reduction of pyocyanin production from *P. aeruginosa* (Samanta et al., 2017).

## Inhibition of Quorum Sensing by Micro Biogenic Titanium Dioxide NPs

Titanium dioxide NPs possess the ability of inhibiting QS and also have a wide range of activities that includes photo catalysis of organic dyes, usage within the photochromic appliances, gas sensors, dye sensitized solar cells, and antimicrobial activities (Banerjee, 2011). It has been observed that TiO_2_NPs in the presence of UV rays produce super-oxides that help in inhibiting the growth of MRSA (Shah et al., 2008). These NPs possess the ability to oxidize the organic substances that are present within the bacterial cells and thus kill the cells (Fujishima et al., 2000; Cho et al., 2005; Shiraishi and Hirai, 2008). Experimental observations indicated that AgCl-TiO_2_ NPs was an effective anti-quorum sensing compound against *C. violaceum* (Naik and Kowshik, 2014). It has also been found that the silver of Ag NPs can prevent the synthesis of violacein which can precisely block the mechanism of QS. Moreover, the inhibition of QS was observed in the absence of oxo-octanoyl homoserine lactone by AgCl-TiO_2_ NPs.

## Inhibition of Quorum Sensing by Micro Biogenic Silicon Oxide NPs

The mechanism of QS was also inhibited significantly using SiO_2_ NPs which are found naturally in the form of quartz that is present as a major element within minerals, rocks, and sands. These NPs are widely used in biomedicines due to their small particle size and biocompatibility (Malvindi et al., 2012). These NPs are predominantly used in healthcare, chemical, cosmetics, composites, energy microelectronics, aerospace, pharmaceutical, and textile industries (Weiss et al., 2006; Santos et al., 2015). The ROS released by these NPs can also cause damage to DNA, resulting in the death of the cell. Hence, these are regarded as useful substances with antimicrobial activities (Tang and Cheng, 2013). NPs coated with various types of organic components were used to inhibit QS and treat resistant microorganisms by removing signaling molecules from the external environment. Research showed that NPs coated with β-cyclodextrin help in the inhibition of AHL‐ dependent QS of *V. fischeri* (Miller, 2015). The study showed that the presence of β-cyclodextrin associated with Si-NP helps in taking up the AHL-molecule from the environment and reduction in bio luminescence. It has been further observed that these NPs were also able to down regulate *LuxA* and *LuxR* genes.

## Inhibition of Quorum Sensing by Micro Biogenic ZnO Nps

ZnO NPs are used largely in the field of dentistry (Tomczak et al., 2009). These NPs possess the ability of utilizing proteins from the environment, thus bringing about inhibition of metabolism and cytotoxicity and hindering various cellular processes (Horie et al., 2009). They exhibit a potent antibacterial property by inhibiting bacterial growth and the adherence property of the cells, thus also preventing the formation of biofilm (Yamamoto, 2001; Brayner et al., 2006). Various research works have been performed that demonstrated the strength of ZnO NPs to act as a potent anti-QS agent. The inhibition of the QS mechanism greatly influenced the biofilm formation in a *P. aeruginosa* strain isolated from Cystic fibrosis (CF; García-Lara et al., 2015). These NPs possess the ability of down regulating the QS genes within the Gram-negative bacterial cells. Researchers showed that ZnO NPs were able to inhibit QS in *P. aeruginosa* by down regulating *lasR*. *lasI*, *rhl I*, and *rhl R* (Saleh et al., 2019). Another study showed that ZnO NPs were able to reduce the swimming and swarming motility within *P. aeruginosa* and also showed its efficacy against the *pqs* and *las* system of QS (Khan et al., 2020). ZnO NPs resulted in the efflux of the zinc cation efflux pump of czc operon and various other regulators of transcription such as type III repressor ptrA and porin gene opdT followed by the repression in the production of pyocyanin-associated phz operon. The ZnO NPs also possess the ability of enhancing membrane hydrophobicity in *P. aeruginosa* (Lee et al., 2014).

## Conclusion

The bacterial cells possess the ability to sense their surrounding population by analyzing the production of auto inducers, known as quorum sensing. This mechanism also helps the bacterial cells to communicate with one another, resulting in the development of biofilm. The mode of action of QS inhibition is mainly *via* inhibition of signal generation, blockage of signal receptors, and disruption of QS signals. Currently, research is focused on finding ways to interrupt the process of bacterial QS using compounds of microbial origin. Microbial biofilm are increasingly causing medical-implant-associated infections. Thus, novel treatment strategies are urgently required for device-associated biofilms infections. The use of nano materials has emerged as a promising approach in preventing biofilm formation by destroying the exopolysaccharides (EPS) of the biofilm matrix and killing the bacteria. Key factors responsible for using nanomaterials for biofilm treatment are their low cytotoxicity and novel mechanisms of action. Nanoparticles’ toxicity strongly depends on their physicochemical properties such as shape, size, surface chemistry, structure, agglomeration state, and cell types in contact with the nano materials (Tran et al., 2020).

The few disadvantages involving microbial nanoparticle synthesis are the tedious purification steps and poor understanding of the mechanisms. Additionally, controlling the shape, size, and mono dispersity in the solution phase is a matter of concern. An important challenge is scaling up the production level processing for industrial applications. This includes addressing a few important issues such as selection of ideal bacteria depending on growth rate, enzyme activities, and biochemical pathways, selection of the biocatalyst state (bacterial enzymes) either of whole cells, crude enzymes, or purified enzymes that could increase the rate of reaction, and optimal conditions for cell growth and enzyme activity. Optimization is also needed for higher biomass synthesis, optimal reaction conditions for better removal of unwanted residual nutrients and metabolites, better extraction and purification processes (freeze-thawing, heating processes, and osmotic shock) of the nanoparticles, and better stabilization of the produced NPs without aggregation (Iravani, 2014).

Microbiologically-synthesized nanoparticles have emerged as a new agent that can be used to either down regulate the operon associated with quorum sensing proteins, or to enhance quorum-quenching activity to prevent biofilm formation. Although QS inhibition shows good potential for treatment of infections, further development and research are necessary to fully understand the mechanisms of action and suitability for clinical applications. Nanomaterial-based treatment methods are expected to continue developing more sophisticated or more complex mechanisms of destroying the EPS and killing the bacteria, although the need for future developments to prevent recurrence after biofilm treatment is still needed.

## Author Contributions

All authors listed have made a substantial, direct and intellectual contribution to the work, and approved it for publication.

### Conflict of Interest

The authors declare that the research was conducted in the absence of any commercial or financial relationships that could be construed as a potential conflict of interest.
